# P-1943. Risk Factors for Coronavirus disease 2019 (COVID-19)-Associated Pulmonary Aspergillosis (CAPA) in Critically Ill Patients: The Predictive Value of KL-6 for CAPA and Mortality

**DOI:** 10.1093/ofid/ofae631.2102

**Published:** 2025-01-29

**Authors:** Seung Min Lee, Si-Ho Kim, Cheon-Hoo Jeon, Yu Mi Wi

**Affiliations:** Samsung Changwon Hospital, Changwon, Kyongsang-namdo, Republic of Korea; Division of Infectious Diseases, Samsung Changwon Hospital, Sungkyunkwan University, Changwon, Kyongsang-namdo, Republic of Korea; Samsung Changwon Hospital, Changwon, Kyongsang-namdo, Republic of Korea; Samsung Changwon Hospital, Changwon, Kyongsang-namdo, Republic of Korea

## Abstract

**Background:**

Coronavirus Disease 2019 (COVID-19) is an acute respiratory viral illness that can be complicated by secondary invasive fungal infections, such as COVID-19-Associated Pulmonary Aspergillosis (CAPA). Krebs von den Lungen-6 (KL-6), a mucinous high-molecular-weight glycoprotein found on the surface of type II alveolar epithelial cells, has been known as a biomarker indicative of respiratory epithelial damage. This study aims to evaluate the risk factors for CAPA in critically ill COVID-19 patients, with a focus on the predictive value of KL-6.

Overall 30-day survival rates between patients with and without CAPA

**Figure:**
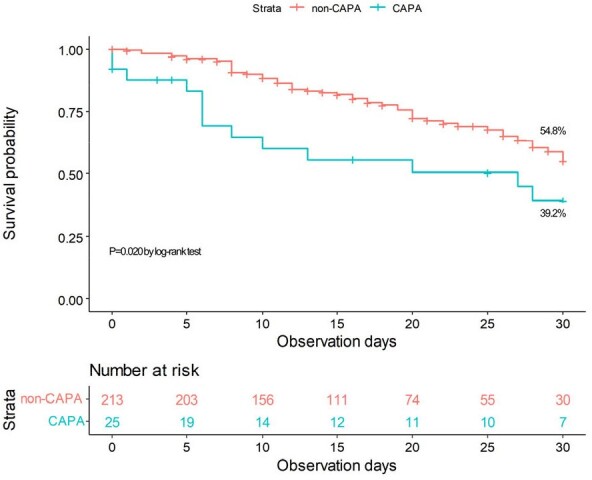

**Methods:**

This single-center retrospective cohort study included critically ill COVID-19 patients (those requiring high-flow respiratory support or mechanical ventilation) admitted from January 2021 through June 2023. CAPA diagnosis was established based on the 2020 European Confederation of Medical Mycology and the International Society for Human and Animal Mycology consensus criteria. The study investigated factors associated with CAPA and compared overall 30-day mortality for patients with and without CAPA.

**Results:**

Among 238 critically ill COVID-19 patients, 25 (10.5%) were diagnosed with CAPA. Notably, patients with CAPA exhibited significantly higher median serum peak levels of KL-6 (787.4 vs. 290.2 U/mL, P< 0.001), with a cutoff value of 392.9 U/mL determined by the Youden index. Multivariable analysis revealed that a peak KL-6 level ≥ 392.9 U/mL was strongly associated with CAPA (adjusted odds ratio 8.62, 95% confidence interval [CI] 2.43-31.69). Additionally, inotropics/vasopressor support, diabetes mellitus, and tocilizumab were identified as factors associated with the development of CAPA. The overall 30-day mortality was significantly higher in patients with CAPA (60.8%) compared to those without (45.2%) (P=0.020), with the difference persisting after adjustment (adjusted hazard ratio 2.06, 95% CI 1.05-4.03). Furthermore, along with older age, lacticemia, and diabetes mellitus, elevated KL-6 was also associated with increased mortality.

**Conclusion:**

Our study suggests that KL-6 may be a significant predictor of both the development of CAPA and mortality among critically ill patients with COVID-19.

**Disclosures:**

All Authors: No reported disclosures

